# Epidemiology of HPV genotypes in Uganda and the role of the current preventive vaccines: A systematic review

**DOI:** 10.1186/1750-9378-6-11

**Published:** 2011-07-12

**Authors:** Cecily Banura, Florence M Mirembe, Anne R Katahoire, Proscovia B Namujju, Anthony K Mbonye, Fred M Wabwire

**Affiliations:** 1 Child Health and Development Centre, Makerere University College of Health Sciences, P. O. Box 6717, Kampala, Uganda; 2 Department of Obstetrics and Gynecology, Makerere University College of Health Sciences, P.O. Box 7072, Kampala Uganda; 3 Uganda Virus Research Institute, P.O. Box 49, Entebbe, Uganda; 4 National Institute for Health and Welfare, Oulu, Finland; 5 Department of Community Health Ministry of Health, P.O. Box 7272, Kampala, Uganda; 6 School of Public Health, Makerere University College of Health Sciences, P.O. Box 7072, Kampala, Uganda

## Abstract

**Background:**

Limited data are available on the distribution of human papillomavirus (HPV) genotypes in the general population and in invasive cervical cancer (ICC) in Uganda. Yet, with the advent of preventive HPV vaccines that target HPV 16 and 18 responsible for causing about 70% of ICC cases in the world, such information is crucial to predict how vaccination and HPV-based screening will influence prevention of ICC.

**Methods:**

To review the distribution of HPV infection and prevalent genotypes, electronic databases (e.g. PubMed/MEDLINE and HINARI) were searched for peer reviewed English articles on HPV infection up to November 30, 2010. Eligible studies were selected according to the following criteria: DNA-confirmed cervical or male genital HPV prevalence and genotypes, HPV incidence estimates and HPV seroprevalence among participants.

**Results:**

Twenty studies were included in the review. Among HIV negative adult women, the prevalence of HR-HPV infections ranged from 10.2% -40.0% compared to 37.0% -100.0% among HIV positive women. Among HIV positive young women aged below 25 years, the prevalence of HR-HPV genotypes ranged from 41.6% -75.0% compared to 23.7% -67.1% among HIV negative women. Multiple infections with non vaccine HR-HPV genotypes were frequent in both HIV positive and HIV negative women. The main risk factors for prevalent HPV infections were age, lifetime number of sexual partners and HIV infection. Incident infections with HR-HPV genotypes were more frequent among adult HIV positive than HIV negative women estimated at 17.3 and 7.0 per 100 person-years, respectively. Similarly, incident HR-HPV among young women aged below 25 years were more frequent among HIV positive (40.0 per 100 person-years) than HIV negative women (20.3 per 100 person-years) women. The main risk factor for incident infection was HIV infection. HPV 16 and 18 were the most common genotypes in ICC with HPV 16/18 contributing up to 73.5% of cases with single infections.

Among uncircumcised adult HIV positive males, HR-HPV prevalence ranged from 55.3% -76.6% compared to 38.6% -47.6% in HIV negative males. Incident and multiple HR-HPV infections were frequent in HIV positive males. Being uncircumcised was the main risk factor for both prevalent and incident HPV infection.

**Conclusion:**

Infections with HR-HPV genotypes were very common particularly among HIV positive individuals and young women irrespective of HIV status. Given the high prevalence of HIV infection, HPV-associated conditions represent a major public health burden in Uganda. However, although the most common HPV genotypes in ICC cases in Uganda were those targeted by current preventive vaccines, there were a large number of individuals infected with other HR-HPV genotypes. Technology allowing, these other HR-HPV types should be considered in the development of the next generation of vaccines.

## Introduction

Invasive cervical cancer (ICC) remains a leading cause of death among women in low-resource settings [1] and virtually every case of ICC is caused by HPV [2]. Genital HPV infection is predominantly, but not exclusively, a sexually transmitted infection and about 50% to 80% sexually active women are infected by this virus at least once in their lifetime [3,4]. Worldwide, women in developing countries account for about 85% of both annual cases of ICC estimated at 493,000 and annual deaths from ICC estimated at 273,500 [1]. Men play an important role in HPV transmission as HPV DNA has been detected in the genitalia of up to 73% of healthy men [5].

Over 100 HPV genotypes have been identified and are divided into high-risk (HR) and low risk (LR) depending on their potential to cause cancer. There are about 15 cancer causing HPV genotypes that are responsible for 5% of all human cancers but predominantly cervical cancer and other anogenital cancers [6]. HPV 16 and HPV 18 are the most virulent HR-HPV genotypes causing about 70% of all ICC in the world [7]. Two preventive vaccines targeting HPV 16 and 18 have been developed and are currently licensed in over 100 countries including Uganda; Cervarix^®^, a bivalent HPV 16/18 from GlaxoSmithKline Biologicals (GSK) and Gardasil^®^, a quadrivalent HPV 6/11/16/18 vaccine from MSD Merck. Gardasil also targets two LR-HPV genotypes 6 and 11, which together cause 90% of genital warts [8]. These current preventive HPV vaccines however, offer protection only against a few cancer causing HPV genotypes.

Uganda has a large burden of ICC. Current estimates indicate that every year, 3,577 women are diagnosed with ICC and 2,464 die from the disease. Compared to the USA, the age adjusted cervical cancer incidence per 100,000 population per year is 8.3 times higher in Uganda (47.5 vs.5.7) and age adjusted death rate as a result of ICC is 20.5 times higher (34.9 vs. 1.7) [9]. The high incidence of ICC is a reflection of prevalent HR-HPV infections and failure to prevent their clinical manifestations by effective screening programs that have dramatically reduced incidence in developed countries [10]. The introduction of Cervarix^® ^by the Program for Appropriate Technology for Health (PATH) in partnership with the Uganda Ministry of Health may offer the greatest hope of significant reduction of ICC burden provided the vaccine is widely and properly delivered [11]. Furthermore, harmonizing primary (vaccination) and secondary prevention (screening) will result in greater reduction of ICC than either program alone [12].

Limited data are however, available on the distribution of HPV genotypes in the general population and in ICC in Uganda. Yet, with the advent of HPV vaccination, such information is crucial for policy makers to predict how HPV vaccination and HPV-based screening will influence prevention of ICC. Moreover, even with a successful vaccination program, vaccinated women will still require screening to detect those that will develop ICC from other HR-HPV genotypes that are not prevented by current vaccines. Therefore, the objective of this review was to summarize published data on HPV infection in Uganda. This review will provide useful baseline information for evaluating the impact of preventive HPV vaccines and HPV based technologies used in screening programs.

## Methods

PubMed/MEDLINE and HINARI websites were searched for peer reviewed English language published medical literature on HPV infection in Uganda up to November 30, 2010. The search was conducted using the following medical subject heading (MESH) and text words either singly or in combination: "HPV", "genotypes"," cervical cancer", "screening", "vaccination", "serology" and "Uganda". Eligible studies were selected according to the following criteria: DNA-confirmed cervical or male genital HPV prevalence, HPV incidence estimates and HPV seroprevalence among participants. From each article, information on first author, publication journal and date, dates of sample collection, HPV detection methods, study design, anatomical site and methods of sample collection, population description, mean or median age of sample with range when available, sample size, the prevalence of HIV when available, the prevalence of HPV (overall, high risk, low risk, specific genotypes) as well as the prevalence of HPV among HIV positive and HIV negative women. Risk factors for HPV infection were also extracted when available.

Twenty studies were found and were included in the review: 9 prevalence studies, 2 prospective cohort studies in women, 3 randomized controlled trials in men, 4 studies on ICC, and 2 seroprevalence studies.

### Methods used for the detection of HPV DNA, typing for specific genotypes and to determine seroprevalence

Various techniques and assays were used for detection of HPV DNA and genotyping. Polymerase Chain Reaction™ (PCR) using generic or consensus primers and Hybrid capture™ 2 (HC2, Digene Co., Gaithersburg, MD, USA) were used in majority of studies. Both techniques have been optimized to detect 13 HR-HPV genotypes [13]. However, some studies used the Southern Blot technique which generally has low sensitivity and detects only a few HR-HPV genotypes. One HPV seroprevalence study was done before the World Health Organization (WHO) developed international standard reagents for calibration of HPV DNA assays and kits [14]. Therefore, caution should be taken when interpreting results from the different studies as the sensitivities and specificities of the HPV assays vary widely depending on the type and quality of the biological specimen and the type and quality of reagents used. Additionally, assays differ in their ability to detect different HPV genotypes particularly where there are co-infections with multiple HPV types [15].

## Results

The findings from studies conducted in selected populations across the country are summarized in Tables 1, 2, 3, 4, 5, 6.

**Table 1:** HPV prevalence and genotype distribution in women with and without cervical abnormalities

Author	Area, subjects	Study design	HPV test	HPV prevalence for any, HR*, LR** and specific genotypes among all tested women^a^or only HPV positive women^b ^	HPV prevalence and type specific genotypes among among all tested women^a^or only HPV positive women^b ^by HIV status	Comments
**Population-Based studies**						
Serwadda et al., 1999 [52]	Rakai district, Random sample of 960 women aged 15-59 years using self collected vaginal swabs	Cross-sectional	Hybrid Capture 2	Any HR-HPV^a^, 16.7%	*HIV status^a^* HIV+, 44.3% HIV-, 10.2%	HIV prevalence^a^, 17.8%
Safaeian et al., 2007 [53]	Rakai district, 606 women, median age 30 years (25-38 years)	Baseline of a population-based cohort study	Hybrid Capture 2/PGMY 09/11 assay	Any HR-HPV^a^, 19% *Type-specific genotypes^a^* HPV 16, 4.5% HPV 52, 4.4% HPV 66, 3.0% HPV 58, 3.0%	*HIV status^a^* *HIV positive* Self-collected, 40% Physician collected, 37% *HIV negative* Self-collected, 15% Physician collected, 16%	HIV prevalence^a^, 15.7%
Asiimwe et al., 2008 [21]	Bushenyi district, 314 women with median age of 27 years (range,18-49) using self collected vaginal swabs	Cross-sectional	Hybrid Capture 2 assay	Any HR-HPV^a^, 17.2%		HIV prevalence^a^, 5.6% (self-reported)
Safaeian et al., 2008b [20]	Rakai district, 926 sexually exprienced women aged 15-49 years, mean age 26 years, who provided at least 3 consecutive self-collected swabs	Population-based cross-sectional study	Hybrid Capture 2/Prototype Roche reverse line blot assay	Any HR-HPV^a^, 19.2% *Type-specific genotypes^a^* HPV 52, 2.2% HPV 16, 2.1% HPV 51, 1.7% HPV 66, 1.5% HPV 68, 1.3%	*HIV status^a^* HIV+, 46.6% HIV-, 14.8%	HIV prevalence^a^, 13.7%
**Clinic-Based Studies**						
*Adult women*						
Blossom et al., 2007 [17]	STI clinic in Kampala, 106 women mean age 26.3 years (range,18-51)	Cross-sectional	Roche PCR/reverse strip assay	Any HPV^a^, 46.2% *Type-specific genotypes^b^* HPV 16/18, 18.4% HPV 52, 14.2% HPV 16, 7.5% HPV 58, 7.5%	*Any HPV type^a^* HIV+, 59.5% HIV-, 39.1% *HR-HPV types^b^* HIV+, 100.0% HIV-, 88.9%	HIV prevalence^a^, 34.9%
*Young women below 25 years*						
Banura et al., 2008a [16]	Clinic for teenagers in Kampala, 1275 sexually active young women median age 20 years (range 12-24)	Baseline of a cohort study	SPF_10_/LIPA PCR assay	Any HPV^a^, 74.6% HR-HPV types^a^, 51.4% *Type-specific genotypes^a^* HPV 52, 13.2% HPV 51, 12.3% HPV 18, 10.7% HPV 16, 10.6% LR-HPV types^a^, 39.8% *LR-HPV genotypes^a^* HPV 6, 15.5% HPV 11, 13.3%	*HIV positive women* Any HPV^a^, 87.8% HR-HPV types, 67.1% *Type-specific genotypes^a^* HPV 16, 18.3% HPV 18, 9.8% HPV 6, 15.9% HPV 11, 17.1% *HIV negative women* Any HPV^a^, 73.2% HR-HPV types^a^, 49.7% *Type-specific genotypes^a^* HPV 16, 10.7% HPV 18, 12.1% HPV 6, 15.3% HPV 11, 12.7%	HIV prevalence^a^, 8.6%
Banura et al., 2008b [18]	Antenatal clinic in Kampala 987 young primiparous pregnant women, median age 19 years (range 14-24)	Baseline of a cohort study	SPF_10_/LIPA PCR assay	Any HPV^a^, 60.0% High-risk types^b^, 43.0% *Type-specific genotypes^b^* HPV 52, 12.1% HPV 51, 8.7% HPV 16, 8.4% HPV 18, 5.8% *LR-HPV genotypes* LR-HPV types^b^, 23% *Type-specific genotypes^b^* HPV 6, 5.5% HPV 11, 3.2%	*HIV positive women* Any HPV^a^, 72.2% *Type-specific genotypes^b^* HPV 16, 18.1% HPV 18, 6.9% HPV 6, 8.3% HPV 11, 1.4% *HIV negative women* Any HPV^a^, 58.9% *Type-specific genotypes^b^* HPV 16, 7.7% HPV 18, 5.7% HPV 6, 5.3% HPV 11, 3.4%	HIV prevalence^a^, 7.3%
**Screened adult women**						
Buonaguro et al., 2000 [54]	Nsambya hospital, Scrapes of 16 women with normal ecto-cervical epithelium and different degrees of cervical epithelial lesions	Cross-sectional	Southern Blot analysis	HPV 16^a^, 12.5%		
Taube et al., 2010 [19]	Post-natal clinic at Mulago hospital, Kampala Cervical exfoliated cells from 196 women, 4-12 weeks post-partum and aged between 18-30 years (mean age for HIV -23 ± 3; HIV + 25.4 ± 3.2)	Cross-sectional	Roche Linear array assay	Any HR-HPV^a^, 49.0% HPV 16/18^a^, 24% *HR-HPV types^a^* HPV 16, 8.2% HPV 18, 3.5% HPV 45, 5.1% HPV 58, 5.1% *LR-HPV types^a^* HPV 6, 2.6% HPV 11, 1.0%	*HIV positive* Any HR-HPV^a^, 77.8% *Type-specific genotypes^a^* HPV 16, 13.9% HPV 18, 5.6% HPV 45, 8.3% HPV 58, 5.6% Other HR-HPV, 47.2% LR-HPV types HPV 6, 5.6% HPV 11, 2.8% *HIV negative* Any HR-HPV^a^, 42.5% *Type-specific genotypes^b^* HPV 16, 6.9% HPV 18, 3.1% HPV 45, 4.4% HPV 58, 5.0% Other HR-HPV, 30.6%	HIV prevalence^a^, 18.0%

*HR = High Risk; **LR = Low Risk

^a ^Denominator consist of all the tested women

^b ^Denominator consist of only HPV positive women

**Table 2:** HPV incidence in women without cervical abnormalities

Author	Area, subjects	Study design	HPV test	HPV incidence rate for any, HR*, LR** and type-specific genotypes among all tested women^a ^per 100 PYs	HPV incidence for any and type-specific genotypes all tested women^a ^per 100 PYs by HIV status	Comments
Safaeian et al., 2008a [24]	Rakai district, rural women, median age 29 years (range, 15-59 years)	Prospective cohort	HC 2/PGMY/09/11 assay	Any HR-HPV^a^, 8.7 *Type-specific genotypes^a^* HPV 16, 1.5 HPV 18, 0.9 HPV 45, 1.1 HPV 51, 1.8 HPV 52, 1.1	*HIV Positive* Any HR-HPV^a^, 17.3 *Type-specific genotypes^a^* HPV 16, 3.2 HPV 18, 2.1 HPV 45, 2.7 HPV 51, 5.1 HPV 52, 1.3 *HIV Negative* Any HR-HPV^a ^, 7.0 *Type-specific genotypes*^a^ HPV 16, 1.2 HPV 18, 0.7 HPV 45, 0.8 HPV 51, 1.2 HPV 52, 1.0	HIV prevalence^a^, 14.0%
Banura et al., 2010 [25]	Teenage clinic in Kampala, 380 young women, median age at baseline 19 years (range, 12-24)	Prospective cohort	SPF_10_/LIPA PCR assay	Any HPV^a^, 30.5 HR-HPV types, 20.9 LR-HPV types, 10.6 HPV 16-related types, 10.8 HPV 18-related types, 5.6 *Type-specific genotypes* *HR-HPV genotypes^a^* HPV 16, 3.3 HPV 18, 2.0 HPV 45, 1.3 HPV 51, 4.2 HPV 52, 3.4 *LR-HPV genotypes^a^* HPV 6, 3.4 HPV 11, 1.9	*HIV Positive* Any HR-HPV^a^, 40.0 *Type-specific genotypes^a^* HPV 16, 6.7 HPV 18, 3.0 *HIV Negative* Any HR-HPV^a^, 29.7 *Type-specific genotypes^a^* HPV 16, 3.0 HPV 18, 2.1	*Cumulative prevalence over 27.5 months^a^* HR-HPV types, 68.2% LR-HPV types, 55.0% HPV 16-related types, 48.6% HPV 18-related types, 28.2%

HR* = High Risk

LR** = Low Risk

^a ^Denominator consist of all the tested women

PYs = Person Years

**Table 3:** HPV prevalence and genotype distribution among invasive cervical cancer cases

Author	Area, subjects	Study design	HPV test	HPV prevalence for any and specific genotypes among all tested women^a^	Comments
Schmauz et al., 1989 [55]	Mulago hospital, cervical tissues from 34 cases and 23 controls	Case Control	Southern Blot analysis	Cases^a^ HPV 16/18, 50% HPV 16, 15% HPV 18, 35% Controls^a^ HPV 18, 4.4%	*Prevalence of HIV*^a^ Cases, 5.9% Controls, 21.7% *Odds of cervical cancer* HPV 18 (OR* = 12.0) HPV16/18 (OR* = 22.0)
Odida et al., 2008 [56]	Mulago, Pathology Department 186 paraffin embedded histologically confirmed archival cervical cancer cases Collected 1968-69, 1970-79, and 1980-89.	Case series	SPF_10_/LIPA	Any HPV^a^, 61.3% *Type-specific genotypes*^a^ HPV 16/18, 73.5% HPV 16, 44.7% HPV 18, 28.8% HPV 45, 9.6%	Types 146 Squamous cell carcinoma 35 adenocarcinoma 3 Adenosquamous 2 undifferentiated types
Odida et al., 2010 [57]	Mulago Hospital, Kampala, 171 pairs of paraffin embedded and freshly frozen tissue samples collected between September 2004 and December 2006	Case series	SPF_10_/LIPA	Any HPV^a^, Frozen tissue, 90.1% *Type-specific genotypes*^a^ HPV 16, 47.4% HPV 18, 19.5% HPV 45, 9.7% HPV 51, 1.9% HPV 52, 1.3% Any HPV^a^, Paraffin embedded tissue, 88.9% *Type-specific genotypes*^a^ HPV 16, 47.4% HPV 18, 23.7% HPV 45, 7.9% HPV 51, 1.3% HPV 52, 1.3%	Multiple HPV genotypes^a^ Frozen tissue, 7.1% Paraffin embedded, 4.6% Single HPV genotypes^a^ Frozen tissue, 92.9% (95% CI^†^: 87.6-96.4) Paraffin embedded, 94.7% (95% CI^†^: 89.9-97.7)

^a ^Denominator consist of all the tested women

CI^† ^= Confidence Interval

OR* = Odds Ratio

**Table 4:** HPV prevalence in males participating in circumcision clinical trials

Author	Area, subjects	Study design	HPV test	HPV prevalence for HR*, and LR** genotypes at enrollment and 24 month follow up in intervention§ and control§§	HPV prevalence for any, HR* and LR** genotypes at baseline and 24-month follow-up	Risk Ratio (RR) and 95% CI† for any, HR*, LR** and multiple HPV infections at baseline and 24-month follow-up
Tobian et al., 2009 [58]	Rakai district, 307 men in intervention and 233 men in control group aged 15-49 years who had detectable beta-globulin or HPV. Preputal cavity sampled	Randomized controlled trial	Roche HPV linear array. HR-HPV genotypes 16, 18,31, 33, 35, 39, 45, 51, 52, 56, 58, 59, 66, and 68 tested	At enrollment *HR-HPV genotypes*^a^ Intervention group, 38.1% Control group, 37.1% At 24 months *HR-HPV genotypes*^a^ Intervention group, 18.0% Control group, 27.9%	Intervention Any HPV genotype^b^ Baseline, 61.9% At 24 months, 35.6% *HR-HPV genotypes*^b^ Baseline, 38.1% At 24 months, 18.0% *LR-HPV genotypes*^b^ Baseline, 47.6% At 24 months, 26.2% Control group Any HPV types^b^ Baseline, 62.6% At 24 months, 51.2% *HR-HPV genotypes*^b^ Baseline, 37.1% At 24 months, 27.9% *LR-HPV genotypes*^b^ Baseline, 48.0% At 24 months, 39.4%	Any HPV genotypes^b^ Baseline, 0.99 (0.81-1.21) At 24 months, 0.70 (0.53-0.91) *HR-HPV genotypes*^b^ Baseline, 1.03 (0.79-1.33) At 24 months, 0.65 (0.45-0.94) *LR-HPV genotypes*^b^ Baseline, 0.99 (0.79-1.25) At 24 months, 0.66 (0.49-0.91)
Serwadda et al., 2010 [59]	Rakai district, uncircumcised HIV positive men aged 15-49 years; 103 men in intervention group and 107 men in control group	Randomized controlled trial	Roche HPV linear array	At enrollment *HR-HPV genotypes*^a^ Intervention group, 72.2% Control group, 76.6% *LR-HPV genotypes*^a^ Intervention group, 85.6% Control group, 83.0% At 24 months *HR-HPV genotypes*^a^ Intervention; 55.3% Control group; 71.7% *LR-HPV genotypes*^a^ Intervention group, 49.4% Control group, 77.4%		At 24 months *RR for HR-HPV geno *types 0.77 (0.62-0.97) *RR for multiple genotypes *0.53 (0.33-0.83)
Gray et al., 2010 [60]	Rakai district, uncircumcised HIV negative men aged 15-49 years; 441 randomized to immediate circumcision (intervention) and 399 delayed circumcision (control)	Randomized controlled trial	Roche HPV linear array	*At enrollment* *HR-HPV genotypes*^a^ Intervention arm, 39.1% Control arm, 38.6%		

HR* = High Risk

LR** = Low Risk

CI^† ^= Confidence Intervals

^a ^Denominator include all women tested

^b ^Denominator include only HPV positive women

^§ ^Intervention refers to circumcision arm of the study

^§§ ^Control refers to non circumcision arm of the study

**Table 5:** HPV incidence in males participating in circumcision clinical trials

Author	Area, subjects	Study design	HPV test	HPV incident cases^c ^and rates^d ^for type-specific genotypes per 100 PYs			Proportions of type-specific incident infections at 24-month follow-up			Risk Ratio (RR) and 95% CI^† ^for HR* and multiple infections
Serwadda et al., 2010 [59]	Rakai district, uncircumcised HIV positive men aged 15-49 years; 103 men in intervention group and 107 men in control group	Randomized controlled trial	Roche HPV linear array	At 24 months HR-HPV incident cases^c ^ Intervention group, 42 cases Control group, 57 cases *Multiple infectionincident cases*^c^ Intervention group, 9.9 cases Control group, 24.7 cases			Incident proportions			At 24 months RR for HR-HPV types = 0.74 (0.54-1.01) RR for multiple infection = 0.04 (0.19-0.84).
								Intervention^d^ (%)	Control^d^ (%)	
							HPV 16 HPV 18 HPV 45 HPV 51 HPV 52	5.8 4.3 4.1 5.4 10.1	14.9 11.1 10.3 13.9 8.2	
Gray, et al. 2010 [60]	Rakai district, uncircumcised HIV negative men aged 15-49 years; 441 men in intervention and 399 men in control group	Randomized controlled trial	Roche HPV linear array	At 24 months *HR-HPV incident cases*^c^ Intervention group, 19.7 cases Control group, 29.4 cases *Multiple infection incident cases^c^* Intervention group, 6.7 cases Control group, 14.8 cases *Type specific incidence rates^d^*						At 24 months RR for HR-HPV types = 0.67 (0.51-0.89) RR for multiple infections = 0.45 (0.28-0.73)
					Intervention	Control				
					Incidence/100 PYs					
				HPV 16 HPV 18 HPV 45 HPV 51 HPV 52	3.6 1.6 1.6 4.0 1.6	4.8 5.3 2.4 5.3 3.6				

HR* = High Risk

CI^† ^= Confidence Intervals

PYs = Person Years

^c ^Denominator include all women tested

^d ^Denominator include women with HR-HPV

**Table 6:** HPV Seroprevalence among women with and without invasive cervical cancer

Author	Area, subjects	Study design	HPV test	HPV seroprevalence for any and type specific genotypes	HPV seroprevalence of any and type specific genotypes among HIV positive women	Comments
Newton, et al., 2004) [27]	Mulago hospital, 191 cervical cancer cases and 336 controls; 15 years and older with a new diagnosis of cancer from the wards or outpatient clinics between 1994 and 1998.	Case Control	HPV L1 VLP/ELISA	*Cases* *Type-specific genotypes*^*a*^ HPV 16, 27% HPV 18, 7% HPV 45, 9% *Controls* Any of 3 HPV types^a^, 17% *Type-specific genotypes*^*a*^ HPV 16, 11% HPV 18, 5% HPV 45, 6%	*HIV Positive Controls* Any of 3 HPV types^a^, 22% *Type-specific genotypes^a^* HPV 16, 16% HPV18, 7% HPV 45, 8%	Antibodies against HPV 16 were significantly associated with cervical cancer OR* = 2.0 (1.2-3.1) The risk increased with increasing anti-HPV 16 antibody titre (P_trend _= 0.01)
Namujju, et al., 2010 [26]	Naguru and Nsambya Health Centers in Kampala, 2,053 women seeking antenatal services; mean age 23 years (range, 14-48)	Cross-sectional	Serology/VLP ELISA (6, 11, 16,18,31, 33, 45)	Any of the 7 selected HPV types^a^, 57%		HIV prevalence^a^, 7.0%

^a^Denominator consist of all tested women

OR* = Odds Ratio

### Prevalence of HPV infections in population-and clinic-based studies of women with and without cervical abnormalities

In women below 25 years, 71.8% of women who were positive for HPV 16 and 18 were also infected with other HR-HPV genotypes [16]. Compared to HIV negative women, HIV positive women had more HR-HPV genotypes detected in cervical/vaginal specimens [16-19] and HSIL lesions [17]. The mean number of HPV genotypes detected was higher among HIV positive compared to HIV negative women (2.8; range 1-9) and 2.1; range 1-10, respectively; [*t*-test, 3.88; p < 0.001]) [16]. Likewise, among young primiparous women, the mean number of HPV types detected among HIV positive women was higher (2.4, range 1-10) than among HIV negative women (1.8, range 1-12) [Student's t-test = 2.79, p = 0.005] [18]. HIV positive women were four times more likely to have abnormal cytology than HIV negative women (43% vs. 11.6%, p < 0.001) [19]. HPV positive women co-infected with HIV had other HR-HPV genotypes that did not include HPV 16 or 18 [16,17].

### Risk factors for prevalent HPV infection

Few studies evaluated risk factors for HPV infections. Lifetime number of sexual partners [16,20]; HIV positivity [16,21]; young age at first sexual intercourse [16] and young age [16,21] were significantly associated with HPV infection. Relative to women in 19-25 age-group, the odds of detecting HR-HPV decreased by 48%, 60%, and 79% among women in 26-30, 31-37, and 38-49 age-group [21]. On average, the odds of detecting HR-HPV decreased by 9% for each-year increase in age. However, HPV was only inversely associated with age (P_trend _0.001) in HIV negative women as the prevalence of HPV among HIV positive women seemed to remain high in all age-groups [20]. Other risk factors found included: abnormal cytology [17,18]; employment in a tertiary sector, concurrent pregnancy, presence of genital warts, and testing positive for *Syphilis, Chlamydia trachomatis *and *Neisseria Gonnorhea *[16]. Smoking cigarettes the previous year was significantly associated with HPV infection after controlling for age, age at sexual debut and HIV status (Adjusted OR = 3.74; 95% CI: 1.15-12.16) [21].

HPV 16 and 18 were the most frequent genotypes in ICC cases. Furthermore, results of pooled data compiled by the International Agency for Research on Cancer (IARC) showed a prevalence of HPV 16/18 of 74.1% (95% CI: 66.4-80.8) [22,23].

### Incident and risk factors for HPV infections among women

HIV positive women were twice as likely to have incident infection compared to HIV negative women [24]. Risk factors for incident HPV infections were HIV positivity [24,25]; young age, many lifetime and recent sexual partners and single women [24,25].

### Risk factor for HPV infection in males participating in circumcision trials

Not being circumcised was the main risk factor for both prevalence and incident HR-HPV infections.

### HPV serology

HPV seroprevalence peaked in women below 20 years of age. The most common HR-HPV serotypes was HPV 33 (23%), HPV 16 (21%), HPV 31 (16%), HPV 45 (11%) and HPV 18 (8%). The seroprevalence of LR-HPV 6, 11 and HPV 6/11 combined was 30%, 26%, and 44%, respectively. Of the 1,118 HPV seropositive women, 53% had antibodies to more than one HPV genotype, 30% had 2 HPV genotypes, 14% had 3 HPV genotypes and 9% had 4-7 genotypes [26]. Antibodies against HPV 16 were significantly associated with ICC (OR = 2.0; 95% CI: 1.2-3.1, p < 0.01) [27]. HPV seoprevalence was higher among HIV positive compared to HIV negative women, 74.0% and 56%, respectively. Multiple antibody positivity was more frequent in HIV positive (53%) than HIV negative women (28%) [26].

## Discussion

To the best of our knowledge, this systematic review represents the first ever to evaluate the burden of HPV infections and ICC in Uganda. It is worth noting that comparison of HPV prevalence and incidence rates across studies was hampered by differences in populations studied, laboratory methods used, and variation in HPV genotypes detected. Nevertheless, consistent with other studies in sub Saharan Africa [28], the review demonstrated a high burden of HR-HPV genotypes in the general population and in ICC. HPV 16 and 18 were the most common genotypes in ICC similar to the global HPV distribution pattern [29]. Mathematical models predict that widespread use of preventive HPV vaccines containing genotypes 16/18 have the potential to reduce deaths from ICC by 50% over several decades [30-32]. However, the efficacy of these vaccines in countries like Uganda with high prevalence of HIV infection, endemic malnutrition, malaria infection and intestinal worm infestation is not yet known. Our review also found that the third most frequent HPV genotype after HPV 16 and 18 among women with ICC was HPV 45, which differed from the worldwide distribution (HPV 16, 18, 58) [9]. Variation in HPV genotype distribution in ICC is not new as it has been observed in other regions of the world. It is hypothesized that host immunogenetic factors and biologic interplay between different HPV genotypes or variants are probably responsible [32]. This variation however, implies that any development of the next generation of multivalent preventive HPV vaccines should contain more HR-HPV genotypes than only 16/18. Based on data contained in this review, multivalent vaccines containing HPV 16/18/45 would potentially prevent approximately 83.1% of ICC in Uganda.

The highest prevalence and incidence rates of HPV infections occurred in young women below 25 years similar to previous studies [23]. It is important to note that Ugandan women tend to marry and become sexually active at a young age and often have older and more sexually experienced partners, which factors would put them at greater risk for HPV infection. The main risk factors for prevalent and incident HPV infections were age, number of lifetime number of sexual partners and HIV infection consistent with other studies [23,33].

HIV positive individuals had a high burden of HPV infection at the time when their life-spans are being prolonged by expanded access to highly active anti-retroviral therapy (HAART) and medical care. Since 2003, the HIV prevalence in Uganda has stabilized at about 6.4% among adults and by the end of 2010; approximately 1.1 million were living with HIV [34]. Women were disproportionately affected accounting for about 57% of the total adults living with HIV [34]. However, the proportion of co-infection with HIV and HPV is not known. Though several studies have consistently shown a high burden of HPV-associated diseases in HIV positive women even in the era of HAART [35], presently, there is limited or noexistent routine screening services for many HIV positive women. There are advantages to providing routine screening to HIV positive women. In Zambia, for example, routine screening prevented one death from ICC for every 46 HIV positive women screened [36]. Presently, there is limited data to support or discourage HPV vaccination of HIV positive individuals. To date, immune response to HPV vaccination among HIV positive individuals using the quadrivalent vaccine is limited to a small study of 120 children aged 7-11 years some of whom used anti retroviral therapy, which was conducted in the USA [37]. The antibodies developed by > 99.5% of the vaccinated children were much lower than in HIV negative historical controls raising concerns of perhaps reduced immunogenicity and efficacy.

In this review, HIV positive women seemed to have multiple and more other HR-HPV genotypes than HPV 16/18 consistent with a previous study where 50% of HIV positive women with HPV 16 and 18 were co-infected with other HR-HPV genotypes [38]. Consequently, it is unclear whether the current vaccines containing only HPV 16 and 18 genotypes may potentially prevent fewer cases of ICC among HIV positive women [39]. Fortunately, the current preventive vaccines have shown cross-protection against some non vaccine HR-HPV genotypes and because of this, it is estimated that wide coverage (> 70%) could potentially prevent up to 71% of ICC [40]. Cross-protection even if limited, may be particularly important in low-resource countries like Uganda where screening programs are limited or nonexistent.

Males had a high burden of HR-HPV infections. Studies have shown that males do not perceive themselves to be susceptible to HPV and do not believe that HPV infection is a severe problem to themselves [41]. However, the role of men as vectors of HR-HPV genotypes that cause ICC has been extensively evaluated in epidemiological studies [42-44]. HPV vaccines have been found to be safe and efficacious in males [45]. However, vaccinating males does not appear to be economical when assessed from the view of ICC. Moreover, the WHO does not recommend routine male vaccination as the primary target of vaccination. Yet, the concept of herd immunity in public health would tend to suggest that vaccinating males would provide a double benefit to females in that the fewer males with HPV, the fewer females will be exposed [46].

Although HPV vaccines hold great promise to reduce ICC-associated mortality and morbidity, it remains unclear whether low-resource countries like Uganda will reap the benefits any time soon. The current high cost of the vaccines, poor health infrastructure and competing health priorities will prevent young girls getting the life saving vaccinations. Uganda's health sector remains considerably under-funded. At less than 10% of total government expenditure in fiscal year 2010/2011, public health expenditure remains far below the Abuja target of 15% that the government of Uganda committed to in 2001 [47,48]. Furthermore, presently, there is no known public sector pricing for the HPV vaccines making it difficult to budget for and as such the details of vaccine implementation and cost coverage remain unknown. Fortunately, the Global Alliance for Vaccines and Immunization (GAVI) pledged to consider HPV vaccines in their investment strategy of 2009-2013 [49]. This may provide the much needed financial help to implement HPV immunization programs in low-resource countries where most of the ICC occur.

For prevention, HPV vaccines must be given before sexual debut to ensure that exposure to HPV has not occurred. Accordingly, the World Health Organization recommended that girls between the ages of 9-13 years should be the primary target of vaccination [50]. Given the financial impossibility of vaccinating all eligible women in the reproductive age, it is vital that primary prevention by HPV vaccination is integrated with education on risk reducing behaviors and secondary prevention programs via screening and treatment of precancerous lesions and ICC. The availability of new and inexpensive screening techniques for rapid identification of HR-HPV may help facilitate the use of HPV testing in low-resource countries [51].

## Conclusions

HR-HPV infections were frequent in both females and males. HR-HPV 16 and 18 targeted by the two licensed preventive HPV vaccines are the most common genotypes found in ICC cases. However, there are a large number of individuals infected with other non vaccine HR-HPV genotypes not targeted by the currently available HPV vaccine preparations, indicating that these vaccines could be less effective and leave women at risk for ICC. Furthermore, given the high prevalence of HIV infection, HPV-associated conditions represent a major public health burden in Uganda. Thus, to further reduce this health burden, any future preventive HPV vaccine(s) should contain more HR-HPV genotypes than only two targeted by the current vaccines.

## Competing interests

The authors declare that they have no competing interests.

## Authors' contributions

CB conceived the study, searched the literature, drafted the manuscript and produced the final tables. MMF, KRA, NBP, MKA & WMF made substantial contributions to the manuscript and contributed to data interpretation. All authors read and approved the final manuscript.
